# Prospective spatial-temporal clusters of COVID-19 in local communities: case study of Kansas City, Missouri, United States

**DOI:** 10.1017/S0950268822000462

**Published:** 2023-03-09

**Authors:** Hadeel AlQadi, Majid Bani-Yaghoub, Siqi Wu, Sindhu Balakumar, Alex Francisco

**Affiliations:** 1 Department of Mathematics and Statistics, University of Missouri-Kansas City, Kansas City, MO 64110, USA; 2 Department of Mathematics, Jazan University, 45142 Jazan, Saudi Arabia; 3 City of Kansas City Health Department, 2400 Troost Ave, Kansas City, MO 64108, USA

**Keywords:** Cluster analysis, COVID-19, disease, pandemic, periodic surveillance, spatial analysis, spatio-temporal analysis

## Abstract

Kansas City, Missouri, became one of the major United States hotspots for COVID-19 due to an increase in the rate of positive COVID-19 test results. Despite the large numbers of positive cases in Kansas City, MO, the spatial-temporal analysis of data has been less investigated. However, it is critical to detect emerging clusters of COVID-19 and enforce control and preventive policies within those clusters. We conducted a prospective Poisson spatial-temporal analysis of Kansas City, MO data to detect significant space-time clusters of COVID-19 positive cases at the zip code level in Kansas City, MO. The analysis focused on daily infected cases in four equal periods of 3 months. We detected temporal patterns of emerging and re-emerging space-time clusters between March 2020 and February 2021. Three statistically significant clusters emerged in the first period, mainly concentrated in downtown. It increased to seven clusters in the second period, spreading across a broader region in downtown and north of Kansas City. In the third period, nine clusters covered large areas of north and downtown Kansas City, MO. Ten clusters were present in the last period, further extending the infection along the State Line Road. The statistical results were communicated with local health officials and provided the necessary guidance for decision-making and allocating resources (e.g., vaccines and testing sites). As more data become available, statistical clustering can be used as a COVID-19 surveillance tool to measure the effects of vaccination.

## Introduction

COVID-19 is an infectious disease caused by SARS-CoV-2, a beta coronavirus with crown-like spikes [1]. It is transmitted by close human contact, and respiratory droplets can cause infection upon entering the body [2]. The disease originated from a seafood market in Wuhan, China, in December 2019. However, the World Health Organization declared this outbreak a pandemic in March 2020 [3].

Over 378 million people have been infected, and 5.67 million have died since the outbreak began. Just in the United States, COVID-19 cases reached over 37 million, with more than 628 000 deaths as of August 2021 [4]. Stricter lockdown measures were enforced across the United States to combat rising infection cases, resulting in a global economic and public health crisis.

The pandemic duration, and the enormous impacts on societies, economies and public health have substantially affected the importance of conducting spatial analysis in understanding the spread of COVID-19 [5]. Spatial methods and Geographic Information System (GIS) software were utilised to track the spread of the disease, identify high-risk patients and hotspot areas, and enable real-time communication with healthcare experts and decision-makers [6]. Several studies examined the spatial methods and tools used to analyse the pandemic's distribution patterns in the first and second halves of 2020 [7–9]. The spatial methods were classified into six thematic groups: spatial models, including spatial clustering and mathematical models; multicriteria analysis; remote detection; data mining and networks; web maps; and volunteered geographic information and public participatory GIS [8, 9].

The space-time scan statistic is among the spatial clustering analysis tools that are popular and powerful techniques to perform geographical disease surveillance [10–13]. Space-time scan statistic can identify clusters of the hardest-hit areas and detect when and where transmission of COVID-19 occurs. Utilizing such a tool is crucial to reducing the chances of another wave, avoiding the rise of small local outbreaks and ultimately controlling the epidemic. In addition, the spatial-temporal analysis will lead to implementing more control measures and establishing testing and vaccination sites in the most affected areas [14–16].

Consequently, significant studies on space-time scan statistics in the spread of COVID-19 have been demonstrated globally at the country level, including the USA [14, 15], India [17], Brazil [18], Italy [19], Singapore [17], South Korea [20], French Alps [21], Germany [22], Sergipe [23] and locally at the city level, for example, Sao Paulo in Brazil [16], Punjab in Pakistan [24], East Java in Indonesia [25], etc.

Of particular interest, Kansas City, Missouri (MO), has become one of the significant hot spots for COVID-19 in the United States due to an increase in the rate of positive COVID-19 test results [26]. To combat the threat, Kansas City's mayor, Quinton Lucas, issued a State of Emergency proclamation on 12 March 2020, which mandated masks and restricted gatherings and congregations of individuals [27]. Kansas City, MO also encourages individuals to stay at least six feet away from others, avoid crowds and poorly ventilated spaces, maintain proper hygiene by washing hands thoroughly, and get vaccinated [27]. Regardless of the efficacy of COVID-19 vaccines, many individuals still have concerns about getting vaccinated. For instance, Missouri's vaccination efforts currently rank 43rd in the country, and only 39% of Missourians are reportedly fully vaccinated [28]. Kansas City, MO, reports that only 37.5% of its population is fully vaccinated [29]. With all efforts to control the pandemic, Kansas City, MO, has observed 79 000 confirmed cases and 897 deaths as of August 2021 [30].

In this paper, our objective is to detect periodic significant space-time clusters of confirmed cases of COVID-19 at the zip code level in Kansas City, MO. The analysis focused on daily COVID-19 cases in four equal periods of 3 months between March 2020 and February 2021. The prospective space-time statistic is useful because it detects active and emerging significant clusters of COVID-19 during the four periods, which can be informative for decision-makers to track the active clusters in space and time, update relative risk (RR) for each location affected by the disease, and detect new emerging clusters. As more data become available, spatial clustering can be used as a COVID-19 surveillance tool.

## Materials and methods

### Study area and COVID-19 data

**Fig. 1.**
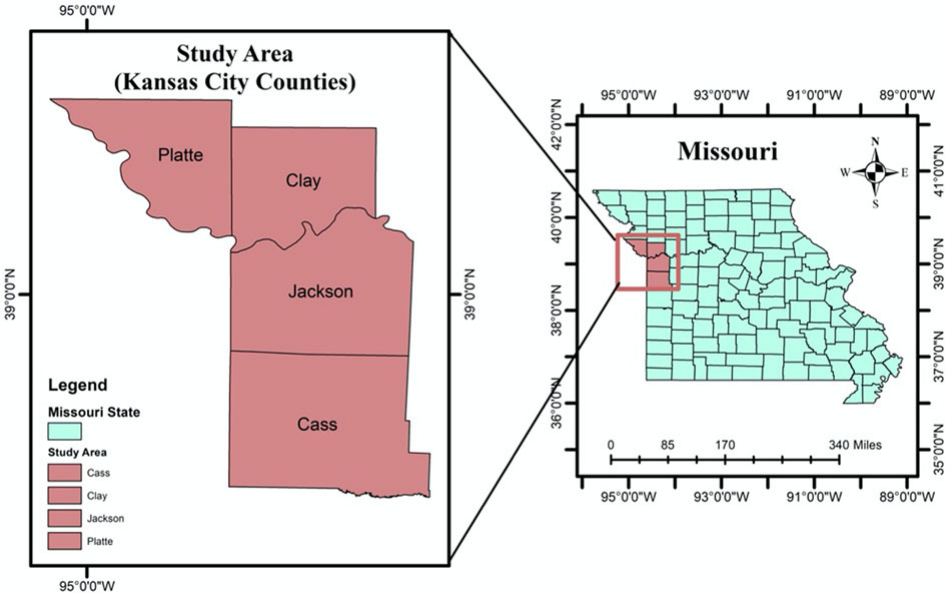
Missouri State map (right), Kansas City counties map (left).

The study area in this research is located in Kansas City, in the western part of the US state of Missouri. Kansas City is located in Jackson County, extending into Clay, Cass and Platte counties [26]. It is located between latitude 39°05′59′′N and longitude 94°34′42′′W (Fig. 1). According to the United States Census Bureau's American Community Survey, Kansas City, MO, had a population of 508 090 in 2020, of which 51.5% were female, 60.9% were white and 23.1% of the total population was under the age of 18 [31]. The space-time study was carried out in each of Kansas City's four counties.

We collaborated with the Kansas City (KCMO) Health Department to obtain the necessary data for this study. The KCMO Health Department provided the daily confirmed cases of COVID-19 between March 2020 and February 2021. This study was approved by the KCMO Health Department, has been granted a waiver of informed consent, and is compliant with the Health Insurance Portability and Accountability Act. We have the same data variables from a previous study [26]. The data contain the following variables: date of case receipt, Epidemiological (Epi) week, Epi year, EpiTrax CMR# [32], age, gender, race, zip code, specimen collection date, vital status and outbreak-associated. For the spatial-temporal prospective study, we need three data files; the first file is the case data, which contain the zip code, the cases in each zip code and the time of each case.

The second data file is the population data, which contain the background of the population (zip code and population in each zip code), and the last data file is the coordinated data that contain the latitude and longitude for each zip code [33].

**Fig. 2.**
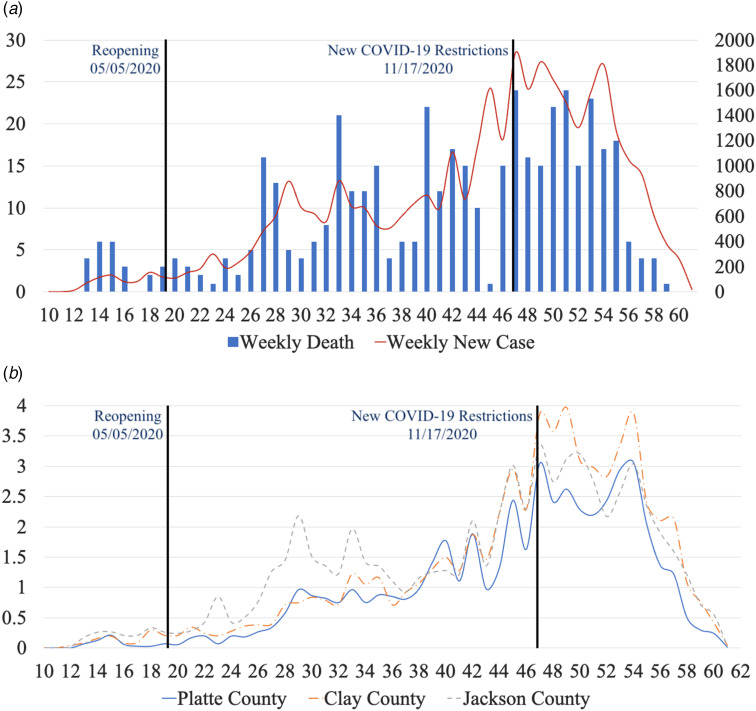
Time-series of COVID-19 cases in Kansas City, MO, between March 2020 and February 2021. (a) Number of new weekly cases and mortality, (b) number of new weekly cases per thousand by county. The vertical lines indicate the dates of reopening and applying the new COVID-19 restrictions.

Table 1 provides the basic descriptive statistics of the Kansas City, MO, weekly COVID-19 data and the weekly COVID-19 cases and deaths until February 2021, as represented in Figure 2(a).

**Table 1.** Descriptive statistics of Kansas City, MO weekly COVID-19 data from March 2020 to February 2021

	# of affected zip codes	Confirmed cases	Mortality
Minimum	10 (0, 3, 7)	13 (0, 6, 7)	0 (0, 0, 0)
Maximum	45 (5, 10, 31)	1899 (216, 617, 1021)	24 (10, 10, 17)
Mean	36.9 (3.8, 8.2, 25.0)	712.9 (71.3, 201.4, 415.2)	9.1 (1.2, 2.3, 5.3)
Median	39 (4, 8, 26.5)	616 (59, 136.5, 383)	6 (0, 1, 3.5)
STD	7.1 (1.0, 1.5, 5.1)	555.9 (65.6, 184.7, 294.6)	7.4 (1.9, 2.6, 5.0)
Range	35 (5, 7, 24)	1886 (216, 611, 1014)	24 (10, 10, 17)

The numbers inside the parentheses correspond to Platte, Clay and Jackson counties, respectively. Of 48 zip codes in Kansas City, MO, there are 5, 11 and 32 zip codes in Platte, Clay and Jackson counties, respectively.

### Space-time method

To detect active or new emerging space-time clusters, we utilised the prospective space-time scan statistic of the discrete Poisson model implemented in SaTScan software developed by Kulldorff *et al.* [34]. Namely, we want to detect space-time COVID-19 clusters in Kansas City that were active and emerging at the end of each study period and ignore the non-statistically significant clusters that may have existed previously but are no longer a public health threat. We utilised the Poisson model to determine the geographical distribution of COVID-19 cases in Kansas City between March 2020 and February 2021, adjusting for the population at risk.

The prospective statistic uses a cylinder window with a space base and a height corresponding to the time. The centre of the cylinder is defined as the centroid of each zip code in Kansas City, MO. The cylinder window was moved through two dimensions: space and time. Hence, we obtained an infinite number of overlapping circles covering the entire study region, and each circle reflected a possible cluster of COVID-19 in the Kansas City area.

To avoid extremely large clusters, we set the upper maximum spatial scanning window size to 10% of the population at risk and the upper maximum temporal bound scanning window size to 50% of each study period. Each cylinder was expanded until the maximum spatial or temporal upper bound is reached. A likelihood ratio test was used to identify the space-time clusters of COVID-19 cases [10]. The likelihood ratio is defined as follows:

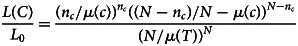

The likelihood ratio was calculated based on the observed and expected number of cases inside and outside that circle, where *L*(*C*) is the maximum likelihood function for cylinder *C*, *L*_0_ is the likelihood function under the null hypothesis, *n_c_* is the number of COVID-19 cases in a cylinder, *µ*(*c*) is the number of expected cases in cylinder *c*, *N* is the total number of all observed cases in Kansas City over time and *µ*(*T*) is the total number of expected cases in Kansas City over time. The likelihood was calculated for each cylinder to determine whether the observed number of cases exceeded the expected number of cases (i.e. the likelihood ratio is >1). The maximum likelihood ratio statistic window constitutes the likeliest cluster (primary cluster). Secondary clusters were also reported if they are statistically significant at the *P*-value < *α* = 0.05.

The results section discusses the statistically significant emerging clusters of COVID-19 in Kansas City at the zip code level in four periods of 3 months: March–May 2020, March–August 2020, March–November 2020 and March 2020–February 2021. The spatial-temporal clusters were analysed using SaTScan™ 9.6, and the maps were plotted using ArcGIS 10.8.

## Results

### Times-series analysis of COVID-19 cases

We used the times-series of COVID-19 cases between March 2020 and February 2021. A total of 1256 cases were eliminated from the data. These cases consisted of 1215 cases from zip codes that did not belong to Kansas City, MO, and 41 cases that were labelled ‘unknown’ in the data (for more details, such as the population of each county based on the zip codes, see Supplementary Tables S1–S4).

Figure 2(a) shows the weekly COVID-19 new cases and mortalities in Kansas City, MO. The total number of cases between March 2020 and February 2021 was 35 647, where 648 cases occurred before the reopening (5 May 2020), and the rest (17 273) after the reopening. After the new COVID-19 restrictions, new weekly cases were stable at first before decreasing continuously. The time-series of the cases (shown with a red curve) had multiple M-shaped (double-top) curves. There was a small ‘double top’ from the 12th week to the 20th week with the maximum values in the 15th and 18th weeks, and the minimum in the 16th week. There were also four ‘double tops’ with larger magnitudes. The first one was from the 27th week to the 37th week, another one from the 37th week to the 43rd week, a third top from the 43rd week to the 48th week and a fourth top from the 48th week to the 55th week. Figure 2(b) shows the weekly cases per thousand for Clay County, Platte County and Jackson County. Before the 16th week, all counties had similar trends in cases per thousand, but Jackson County had more cases per thousand than Clay County and Platte County. Between the 22nd and 37th weeks, Jackson County had more cases per thousand. Between the 39th and 40th weeks, Platte County had more cases per thousand. From the 41st week to the 47th week, all counties had the same trend of cases per thousand, but Platte County had fewer cases per thousand than Clay County and Jackson County. Clay County had more cases per thousand from the 47th week to the 49th week and from the 51st week to the 54th week. After the 54th week, cases per thousand in all counties decreased continuously.

### Space-time clusters of COVID-19 from periods 1 to 4

**Fig. 3.**
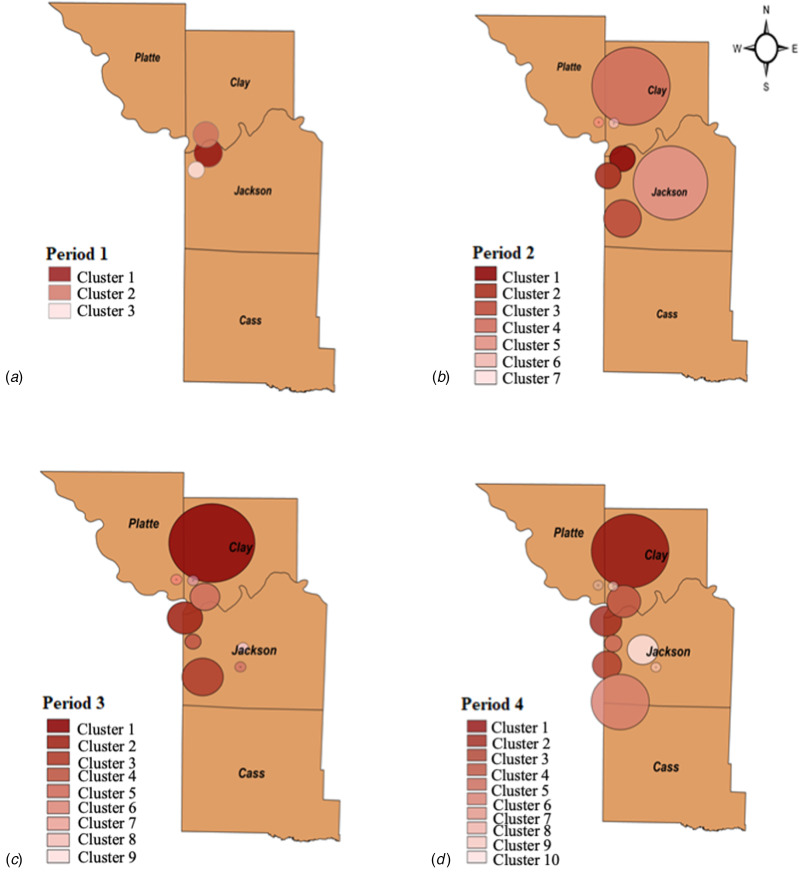
The emergence of COVID-19 clusters in Kansas City during four periods of 3 months: (a) period 1: March–May 2020, downtown Kansas City, MO; (b) period 2: March–August 2020, additional clusters in the north and south; (c) period 3: March–November 2020, further spread of clusters; and (d) period 4: March 2020–February 2021, active clusters along the state line.

Figure 3(a) and Supplementary Table S5 present the characteristics of the three statistically significant emerging space-time clusters of COVID-19 in Kansas City at the zip code level from March to May 2020. During period 1, three statistically significant clusters emerged, mainly concentrated in downtown Kansas City, MO. Cluster 1 was located in northeast Kansas City and contained seven locations in Jackson County, with an RR of 9.21 (expected cases = 37.24; observed cases = 274). Cluster 2 contained two locations in Clay County, where the RR is 5.16, and there are 93 observed cases. Cluster 3 was located in downtown Kansas City, MO, with three locations in Jackson County, where the RR is 2.25, and there are 42 observed cases.

Figure 3(b) and Supplementary Table S6 show the characteristics of seven statistically significant emerging space-time clusters of COVID-19 in Kansas City at the zip code level from March to August 2020. By adding updated data of COVID-19 in Kansas City, four more clusters have emerged. The likeliest cluster (primary cluster) contained eight locations in Jackson County with RR = 5.09. Cluster 2 was located in downtown Kansas City, MO, with six locations in Jackson County and RR = 3.33. Cluster 3 was found in south Kansas City and includes six locations in Jackson County with RR = 3.24. Cluster 4 contained nine locations in Clay County and two locations in Platte County with RR = 2.76. Cluster 5 contained eight locations in Jackson County with RR = 1.71. Clusters 6 and 7 contained only one location, with RR = 1.99 and 1.50 in Platte County and Clay County, respectively. We noticed the disease clusters spread over a wider region in downtown and north of Kansas City. Also, cluster 2 in period 1 disappeared during period 2.

Figure 3(c) and Supplementary Table S7 show the characteristics and spatial patterns of the third period at the zip code level from March to November 2020. During this period, nine statistically significant space-time clusters of COVID-19 were emerging in large areas of north and downtown Kansas City. All clusters emerged during period 2 were still active in period 3. Note that cluster 2, which disappeared during period 2, re-emerged during period 3 as cluster 5. Also, cluster 4 during period 2 became the primary cluster during period 3.

The likeliest cluster (primary cluster) contained nine locations in Clay County and two locations in Platte County, with RR = 3.17. Cluster 2 was located in downtown Kansas City, MO, with eight locations in Jackson County and RR = 2.67. Cluster 3 was found in south Kansas City and includes six locations in Jackson County with RR = 2.13. Cluster 4 contained three locations in Jackson with RR = 2.07. Cluster 5 contained four locations in Clay County with RR = 1.51. Clusters 6 and 9 in Jackson County contained one location each with RR = 3.88 and 2.16, respectively. Clusters 7 and 8 contained one location each, with RR = 1.58 and 1.49 in Platte County and Clay County, respectively.

Figure 3(d) and Supplementary Table S8 show the locations and spatial patterns of the spread of the disease in the fourth period from March 2020 to February 2021. Ten clusters were present, and the most active clusters were along the State Line Road. Note that all clusters in period 3 were still active during the fourth period.

The primary cluster contained nine locations in Clay County and two locations in Platte County with RR = 3.18. Cluster 2 was located in downtown Kansas City, MO, with eight locations in Jackson County and RR = 2.19. Cluster 3 was a new cluster that emerged in this period, containing three locations with RR = 1.95 in Jackson County. Cluster 4 contained four locations in Clay County and one location in Jackson County with RR = 2.23. Cluster 5 contained four locations in Jackson County with RR = 1.93. Cluster 6 contained five locations in Jackson County and one location in Cass County with RR = 1.87. Clusters 7, 8 and 9 contained one location each, with RR = 2.34, 4.83 and 1.51 in Platte County, Jackson County and Clay County, respectively. Cluster 10 contained three locations in Jackson County, with RR = 1.22.

By adding updated COVID-19 data, we were able to identify emerging clusters, which allowed us to track the previously detected clusters to determine whether they were growing or shrinking. Table 2 summarises the cluster changes during the four periods, starting with three clusters and ending with 10 clusters at the end of February 2021. For instance, the primary cluster during period 1 with RR = 9.21 shrank during period 2 with RR = 5.09, then grew as cluster 2 during periods 3 and 4 with RR = 2.67 and 2.19, respectively. Also, cluster 2 with RR = 5.16 during period 1 disappeared in period 2 and reappeared with a growing magnitude as clusters 5 and 4 during periods 3 and 4 with RR = 1.51 and 2.23, respectively. In addition, cluster 4 during period 2 with RR = 2.76 became the primary cluster during periods 3 and 4 with RR = 3.17 and 3.18, respectively. Lastly, cluster 5 during period 2, with RR = 1.71 was split into clusters 6 and 9 in period 3, with RR = 3.88 and 2.16, respectively.

**Table 2.** Tracking the number of emerging clusters during the four periods of March–May 2020, with three active clusters, March–August 2020 increased to seven clusters, March–November 2020 with nine active clusters and the last period March 2020–February 2021 reaching to 10 active clusters

Period 1			Period 2			Period 3			Period 4		
Cluster #	RR	Radius (km)	Cluster #	RR	Radius (km)	Cluster #	RR	Radius (km)	Cluster #	RR	Radius (km)
1	**9**.**21**	14	1	5.09	13.25	2	2.67	16.55	2	2.19	16.55
2	5.16	12.56	Disappeared	–	0	5	**1**.**51**	13.94	4	**2**.**23**	17.49
3	2.25	8.10	2	3.33	13.28	4	2.07	7.54	5	1.93	9.11
–	–	–	3	3.24	19.34	3	2.13	19.34	6	1.87	30.21
–	–	–	4	2.76	40.16	1	**3**.**17**	40.16	1	**3**.**18**	40.16
–	–	–	5	1.71	38.34	6 9	**3.88** **2.16**	10.11 10.11	8 10	4.83 1.22	10.11 16.11
–	–	–	6	1.99	15.17	7	1.58	15.17	7	2.34	15.6
–	–	–	7	1.50	15.17	8	1.49	15.17	9	1.51	15.17
–	–	–	–	–	–	–	–	–	3	1.95	15.17

The relative risk (RR) and radius indicate the magnitude of each cluster.

## Discussion

The main point of the prospective approach is the ability to add updated data to identify other emerging clusters and track the risk of previously detected clusters.

In this paper, we utilised a prospective space-time analysis to detect emerging clusters of COVID-19 in Kansas City, MO, at the zip code level, which provided results at four distinct periods: March–May 2020, March–August 2020, March–November 2020 and March 2020–February 2021. By identifying the statistically significant emerging space-time clusters, we could determine regions with increased risk of occurrence. Also, we could measure growth or decay of clusters. The results of this study were shared with the KCMO Health Department to track outbreaks and take necessary steps in the regions with higher risk of COVID-19 infection.

Rapid detection of clusters can help determine whether current preventive and control policies are effective. The prospective strategy used in this study has helped the KCMO Health Department quickly identify hot spots and allocate more resources to those regions to prevent similar outbreaks in the same regions. These measures may include adding more COVID-19 vaccination clinics or COVID-19 testing sites in high-risk areas to help control the severity and spread of the disease. Encouraging social distancing or enforcing mask mandates in these counties/regions could also prevent the spread of COVID-19. Informing public health decision-makers with surveillance data can help them better address the needs of underserved areas and promote a safer, healthier community.

Prospective space-time analysis detected three statistically significant clusters that emerged during March–May 2020, which were mainly concentrated in downtown Kansas City. By adding updated data of COVID-19 in Kansas City from March to August 2020, the emerged clusters increased to seven clusters that were spreading across a broader region in downtown and north of Kansas City. The result of adding more data was nine clusters covering large areas of north and downtown Kansas City, and 10 clusters were present and further extended the infection along the State Line Road during the two distinct periods: March–November 2020 and March 2020–February 2021, respectively.

We also observed temporal variations in the number of COVID-19 cases between March 2020 and February 2021. Compared to the weekly cases per thousand for Clay County and Platte County, Jackson County had the most significant cases per thousand for the first 34 weeks. However, after the 37th week, the COVID-19 outbreak turned, and Clay County took the lead.

Despite the strength of completing a prospective study in Kansas City, MO, some limitations exist. First, to understand why COVID-19 has severely impacted specific locations, it is essential to adjust the study for relevant factors such as socio-economic status, demographic factors, human mobility, tourism, religious gatherings, economic networks, etc. These factors may impact the spread of COVID-19 across communities. For instance, in a previous study, it was observed that demographic factors may impact the geographical distribution of COVID-19 cases in Kansas City, MO [26]. By conducting retrospective spatial analysis, the researchers found significant differences in COVID-19 clusters with respect to demographic factors of gender, race and ethnicity. Hispanic populations had the most scattered clusters concentrated in downtown Kansas City, MO, and the highest prevalence of COVID-19 cases compared to African American and White populations. In a different study, tourism was associated with a high number of confirmed cases of COVID-19 in northeastern Brazil [35]. In Wuhan, China, human mobility was associated with the high transmission of SARS-CoV-2, and social distancing policies effectively controlled the epidemic [36]. In Ecuador, economic networks and commercial flow played a significant role in spreading the COVID-19 in the region [37]. In Malaysia, the substantial spread of COVID-19 cases was linked to a religious gathering held without adhering to the social distancing recommendation [38]. Therefore, it is crucial to study the different factors that impact and are associated with the spread of COVID-19 in the communities.

Second, our surveillance focused on the pandemic before vaccine distribution, so trends and emerging clusters may change over time with the extension of the current data. Therefore, completing the space-time analysis over an extended period will provide a more reliable tool for surveillance. Third, our data included only confirmed COVID-19 cases obtained from the KCMO Health Department, so we could not gather any information on unconfirmed or probable cases. As a result, a complete perspective on the impact of COVID-19 in Kansas City, MO, is still inconceivable.

Future research can utilise updated data of COVID-19 with respect to the above-mentioned factors to improve the surveillance and detection of emerging clusters. With the availability of updated data and the capabilities of space-time perspective analysis, we can evaluate existing measures for limiting the spread of COVID-19 variants and forecast future outbreaks of infectious disease.

## Data Availability

The data that support the findings of this study are available on request from KCMO Health Department. The data are not publicly available due to their containing information that could violate HIPAA confidentiality requirements.

## Supplementary material

For supplementary material accompanying this paper visit http://doi.org/10.1017/S0950268822000462. click here to view supplementary material
