# Supplementary material for: Prospective spatial-temporal clusters of COVID-19 in local communities: case study of Kansas City, Missouri, United States
Source: Epidemiol Infect. 2023 Mar 9;151:e178. doi: 10.1017/S0950268822000462 (PMC10600737; doi:10.1017/S0950268822000462)
Supplement: Supplementary file 1 [file S0950268822000462sup001.docx]

*Epidemiology and Infection*

**Prospective Spatial–Temporal Clusters of COVID-19 in Local Communities: Case Study of Kansas City, Missouri, United States**

Hadeel Alqadi^1,2,*^, [hadz6@mail.umkc.edu](mailto:hadz6@mail.umkc.edu)

Majid Bani Yaghoub^1^, [baniyaghoubm@umkc.edu](mailto:baniyaghoubm@umkc.edu)

Siqi Wu^1^, [sw4fc@mail.umkc.edu](mailto:sw4fc@mail.umkc.edu)

Sindhu Balakumar^1^, [sbmbg@mail.umkc.edu](mailto:sbmbg@mail.umkc.edu)

Alex Francisco^3^, [Alex.Francisco@kcmo.org](mailto:Alex.Francisco@kcmo.org)

^1^ Department of Mathematics and Statistics

University of Missouri-Kansas City

Kansas City, Missouri 64110-2499, USA

^2^ Department of Mathematics, Jazan University, Saudi Arabia

^3^ City of Kansas City, Missouri Health Department

2400 Troost Ave Kansas City, MO 64108

**Table S1.** Populations of Platte County, Clay County, and Jackson County in Kansas City, Missouri. These populations were collected based on zip code.

| County | Platte | Clay | Jackson | total |
| --- | --- | --- | --- | --- |
| Population | 70,574 | 155,551 | 304,558 | 530,683 |

**Table S2.** Population of Platte County, Missouri based on the zip codes.

| zip code | population |
| --- | --- |
| 64151 | 26,128 |
| 64152 | 28,307 |
| 64153 | 5,383 |
| 64154 | 10,497 |
| 64164 | 259 |

**Table S3.** Population of Clay County, Missouri based on the zip codes.

| zip code | population |
| --- | --- |
| 64116 | 15,372 |
| 64117 | 15,001 |
| 64118 | 42,493 |
| 64119 | 26,830 |
| 64155 | 23,108 |
| 64156 | 7,372 |
| 64157 | 19,680 |
| 64158 | 4,796 |
| 64165 | 197 |
| 64166 | 225 |
| 64167 | 477 |

**Table S4.** Population of Jackson County, Missouri based on the zip codes.

| zip code | Population |
| --- | --- |
| 64101 | 279 |
| 64102 | 0 |
| 64105 | 4,306 |
| 64106 | 9,360 |
| 64108 | 7,491 |
| 64109 | 9,188 |
| 64110 | 16,745 |
| 64111 | 17,398 |
| 64112 | 8,698 |
| 64113 | 12,120 |
| 64114 | 24,760 |
| 64120 | 297 |
| 64123 | 9,017 |
| 64124 | 10,534 |
| 64125 | 1,414 |
| 64126 | 6,488 |
| 64127 | 14,707 |
| 64128 | 12,056 |
| 64129 | 8,898 |
| 64130 | 20,215 |
| 64131 | 22,485 |
| 64132 | 14,474 |
| 64134 | 23,836 |
| 64136 | 2,211 |
| 64137 | 11,349 |
| 64138 | 26,485 |
| 64139 | 1,925 |
| 64145 | 5,288 |
| 64146 | 1,574 |
| 64147 | 640 |
| 64149 | 320 |
| 64192 | 0 |

Populations for each zip code were collected from [www.*bestplaces.net*](http://www.bestplaces.net)

We used the following resources to find the population of each county based on the zip codes.

Population of Platte County: <https://www.bestplaces.net/find/zip.aspx?county=29165&st=MO>

Population of Clay County: <https://www.bestplaces.net/find/zip.aspx?county=29047&st=MO>

Population of Jackson County: <https://www.bestplaces.net/find/zip.aspx?county=29095&st=MO>

**Space-time clusters of COVID-19 from periods 1 to 4**

Table S5: Emerging space-time clusters of COVID-19 March–May 2020 (Period 1) at the zip code level

| Cluster | Duration (Month) | RR* | Observed | Expected | Counties | # of locations | *P*-value |
| --- | --- | --- | --- | --- | --- | --- | --- |
| Cluster 1 (Primary) | May | 9.21 | 274 | 37.24 | Jackson County | 7 | 1*10^-17^ |
| Cluster 2 | May | 5.16 | 93 | 19.20 | Clay County | 2 | 1*10^-17^ |
| Cluster 3 | May | 2.25 | 42 | 19.06 | Jackson County | 3 | 2.4*10^-4^ |

Table S6: Emerging space time clusters of COVID-19 March–August 2020 (Period 2) at the zip code level

| Cluster | Duration (Month) | RR* | Observed | Expected | Counties | # of locations | *P*- value |
| --- | --- | --- | --- | --- | --- | --- | --- |
| Cluster 1 (Primary) | June-Aug | 5.09 | 1717 | 403.71 | Jackson County | 8 | 1*10^-17^ |
| Cluster 2 | July-Aug | 3.33 | 802 | 258.17 | Jackson County | 6 | 1*10^-17^ |
| Cluster 3 | July-Aug | 3.24 | 825 | 273.68 | Jackson County | 6 | 1*10^-17^ |
| Cluster 4 | July-Aug | 2.76 | 694 | 265.27 | Clay County  Platte County | 11 | 1*10^-17^ |
| Cluster 5 | July-Aug | 1.71 | 367 | 218.11 | Jackson County | 8 | 1*10^-17^ |
| Cluster 6 | July-Aug | 1.99 | 214 | 108.71 | Platte County | 1 | 1*10^-17^ |
| Cluster 7 | Aug | 1.50 | 135 | 90.52 | Clay County | 1 | 1.4*10^-3^ |

Table S7: Emerging space time clusters of COVID-19 March–November 2020 (Period 3) at the zip code level

| Cluster | Duration (Month) | RR* | Observed | Expected | Counties | # of locations | *P*-value |
| --- | --- | --- | --- | --- | --- | --- | --- |
| Cluster 1 (Primary) | Aug-Nov | 3.17 | 2135 | 735.21 | Clay County  Platte County | 11 | 1*10^-17^ |
| Cluster 2 | Aug-Nov | 2.67 | 1808 | 724.52 | Jackson County | 8 | 1*10^-17^ |
| Cluster 3 | Aug-Nov | 2.13 | 1541 | 758.52 | Jackson County | 6 | 1*10^-17^ |
| Cluster 4 | Aug-Nov | 2.07 | 1098 | 548.78 | Jackson County | 3 | 1*10^-17^ |
| Cluster 5 | Aug-Nov | 1.51 | 1053 | 712.18 | Clay County | 4 | 1*10^-17^ |
| Cluster 6 | Aug-Nov | 3.88 | 85 | 22.00 | Jackson County | 1 | 1*10^-17^ |
| Cluster 7 | Aug-Nov | 1.58 | 472 | 301.30 | Platte County | 1 | 1.3*10^-15^ |
| Cluster 8 | Oct-Nov | 1.49 | 371 | 250.88 | Clay County | 1 | 3.4*10^-9^ |
| Cluster 9 | Aug-Nov | 2.16 | 58 | 26.88 | Jackson County | 1 | 1.4*10^-4^ |

Table S8: Emerging space time clusters of COVID-19 March 2020–February 2021 (Period 4) at the zip code level

| Cluster | Duration (Month) | RR* | Observed | Expected | Counties | # of locations | *P*-value |
| --- | --- | --- | --- | --- | --- | --- | --- |
| Cluster 1 (Primary) | Sept 20- Feb 21 | 3.18 | 4258 | 1475.55 | Clay County  Platte County | 11 | 1*10^-17^ |
| Cluster 2 | Sept 20- Feb 21 | 2.19 | 3020 | 1454.10 | Jackson County | 8 | 1*10^-17^ |
| Cluster 3 | Oct 20-Feb 21 | 1.95 | 2295 | 1221.43 | Jackson County | 3 | 1*10^-17^ |
| Cluster 4 | Dec 20-Feb 21 | 2.23 | 1551 | 715.20 | Clay County  Jackson County | 5 | 1*10^-17^ |
| Cluster 5 | Oct 20-Feb 21 | 1.93 | 2160 | 1155.63 | Jackson County | 4 | 1*10^-17^ |
| Cluster 6 | Oct 20-Feb 21 | 1.87 | 1541 | 844.57 | Jackson County  Cass County | 6 | 1*10^-17^ |
| Cluster 7 | Dec 20-Feb 21 | 2.34 | 694 | 300.68 | Platte County | 1 | 1*10^-17^ |
| Cluster 8 | Oct 20-Feb 21 | 4.83 | 177 | 36.83 | Jackson County | 1 | 1*10^-17^ |
| Cluster 9 | Dec 20-Feb 21 | 1.51 | 748 | 500.73 | Clay County | 1 | 1*10^-17^ |
| Cluster 10 | Dec 20-Feb 21 | 1.22 | 929 | 762.81 | Jackson County | 3 | 1.9*10^-6^ |
